# Making science public: a review of journalists’ use of Open Access research

**DOI:** 10.12688/f1000research.133710.2

**Published:** 2024-01-02

**Authors:** Alice Fleerackers, Natascha Chtena, Stephen Pinfield, Juan Pablo Alperin, Germana Barata, Monique Oliveira, Isabella Peters

**Affiliations:** 1 Scholarly Communications Lab, Simon Fraser University, Vancouver, British Columbia, Canada; 2 Interdisclipinary Studies, Simon Fraser University, Vancouver, British Columbia, Canada; 3 School of Publishing, Simon Fraser University, Vancouver, British Columbia, Canada; 4 Information School, University of Sheffield, Sheffield, UK; 5 Laboratory of Advanced Studies in Journalism, Universidade Estadual de Campinas, Campinas, State of São Paulo, Brazil; 6 ZBW – Leibniz Information Center for Economics, Kiel, Schleswig-Holstein, Germany

**Keywords:** Open science, journalism, COVID-19, open access, preprints

## Abstract

Science journalists are uniquely positioned to increase the societal impact of open research outputs by contextualizing and communicating findings in ways that highlight their relevance and implications for non-specialist audiences. Yet, it is unclear to what degree journalists use open research outputs, such as open access publications or preprints, in their reporting; what factors motivate or constrain this use; and how the recent surge in openly available research seen during the COVID-19 pandemic has affected this. This article examines these questions through a review of relevant literature published from 2018 onwards—particularly literature relating to the COVID-19 pandemic—as well as seminal articles outside the search dates. We find that research that explicitly examines journalists’ engagement with open access publications or preprints is scarce, with existing literature mostly addressing the topic tangentially or as a secondary concern, rather than a primary focus. Still, the limited body of evidence points to several factors that may hamper journalists’ use of these outputs and thus warrant further exploration. These include an overreliance on traditional criteria for evaluating scientific quality; concerns about the trustworthiness of open research outputs; and challenges using and verifying the findings. We also find that, while the COVID-19 pandemic encouraged journalists to explore open research outputs such as preprints, the extent to which these explorations will become established journalistic practices remains unclear. Furthermore, we note that current research is overwhelmingly authored and focused on the Global North, and the United States specifically. We conclude with recommendations for future research that attend to issues of equity and diversity, and more explicitly examine the intersections of open access and science journalism.

## Introduction

Open science (OS) is a global movement aiming to “make scientific research from all fields accessible to everyone” ( UNESCO, 2023). It encapsulates a range of practices that seek to provide free and unrestricted access to research findings (i.e., publishing research papers in publicly available venues) but also to the research process itself (e.g., sharing software, code, protocols, or datasets used in research). Collectively, these practices are united by a vision of a scientific system that is more collaborative, equitable, sustainable, and beneficial—to scientists as well as the wider societies within which they work (ibid.). In line with this vision, an increasing number of scholarly publications are made freely available to the public each year ( Piwowar *et al*., 2018, 2019). Adding to this growth in open access (OA) journal publications is the increasingly common practice of making research freely available ahead of journal peer review in the form of preprints ( Puebla *et al*., 2022). The scholarly community’s use of open research outputs has further accelerated during the COVID-19 pandemic, with an unprecedented number of OA publications and preprints becoming available ( Fraser *et al*., 2021; Waltman *et al*., 2021).

However, making research outputs openly available does not automatically make them accessible to a public audience. Academic publications are written for peer researchers and academics rather than the general public and use the jargon, rhetorical features, and communication norms and conventions of the disciplines within which they are produced ( Fahnestock, 1986). Such specialist language can enhance understanding within these disciplinary communities, contributing to more economical, precise communication that supports collaboration among experts ( Hirst, 2003). However, it can be very difficult for ‘lay’ readers to understand. Thus, realistically, open licensing only expands access to academic and practitioner audiences who have the educational or professional background to read research. For the public to truly engage with and benefit from open outputs, it is necessary to provide not only “technical” or “material” access to research but also “conceptual access” that enables them to understand and use the findings ( Kelly & Autry, 2013).

Science journalists are ideally positioned to provide such conceptual access because they can critique, contextualize, and communicate findings from open research outputs in ways that highlight their relevance and implications for non-specialist audiences. That is, science journalists can help align the ideals of OS “with the realities of complex, specialized genres of writing to provide better, more ‘open,’ access to research” ( Kelly & Autry, 2013, p. 1). Yet, it is unclear to what degree journalists use the resources and outputs emerging as a result of the adoption of OS in their reporting, what factors motivate or constrain this use, and how the recent surge in openly available research seen during the COVID-19 pandemic has affected the relationship between OS and science journalism (SJ) ( Schultz, 2023).

To examine these gaps, we conducted a review of peer-reviewed publications, preprints, editorials, commentaries, and blog posts exploring the intersections of SJ and OS, with a focus on journalists’ use of openly available research outputs (i.e., OA publications and preprints). We focused on these two forms of OS because journalists tend to report on study results, rather than the methods, protocols, or datasets used to conduct the research ( Matthias *et al*., 2019). Using relevant keywords, we searched Google Scholar for literature published since 2018—particularly literature relating to the COVID-19 pandemic—but also included seminal articles (i.e., those frequently mentioned by other sources) outside the search dates. Although Google Scholar indexes literature from many languages, the search algorithm is highly biased towards English-language publications ( Rovira *et al*., 2021); as such, this language bias is a limitation of our review. In addition, relying on Google Scholar likely excluded relevant grey literature, such as policy papers, reports, working papers, and writing by journalists. We extracted, grouped, and abstracted results and arguments using an adapted qualitative metasummary approach ( Sandelowski & Barroso, 2007) to provide a narrative synthesis of the key findings. We found very little scholarship that explicitly examines how OS practices, values, or concepts interface with journalistic ones, nor how journalists engage with open research outputs. Therefore, this review mainly covers research and theoretical contributions that discuss the intersections of OS and SJ tangentially or as a secondary concern, rather than a primary focus. Journalists’ use of open data and open code, while relevant to this discussion, is outside the scope of this paper and will be discussed in future work.

Our findings show that although science journalists are ideally positioned to facilitate public access to research, their potential to do so is hampered by an overreliance on traditional criteria for evaluating scientific quality; concerns about the trustworthiness of open research outputs; and challenges identifying, using, and verifying the findings. We also found that, although the COVID-19 pandemic encouraged journalists to explore OA outputs such as preprints, the extent to which these explorations will become established journalistic practices remains unclear. Additionally, most of the literature reviewed is authored and focused on the Global North, and the United States specifically. In general, more perspectives from and on the Global South are needed, as are empirical studies to be used as an evidentiary base. We conclude with recommendations for future research that is empirically and theoretically grounded, attends to issues of equity and diversity, and more explicitly examines the intersections of OS and SJ.

## The argument for OS-based journalism

Philosopher of science Kevin Elliott is one of few scholars who has explicitly examined the intersection of OS and SJ. In 2019, he proposed that “bringing open science and science journalism into conversation with each other” ( Elliott, 2019, p. 5) could lead to more critical science media coverage that helps audiences better understand the value judgments that shape scientific work. Such critical coverage would move beyond simply reporting research findings to illuminating the process of science itself. In doing so, it could address value judgments inherent in all research—such as the choice of research questions or methods, and the impacts of those choices for the results and their interpretations—but could also focus on those specific to the OS movement, such as the factors that motivate researchers to post articles ahead of peer review (i.e., preprints) or publish in OA journals ( Elliott, 2019). It could also emphasize personally or societally relevant aspects of research findings ( Elliott, 2022), which sometimes differ from those seen as scientifically relevant ( Elliott & Resnik, 2019). Besançon *et al*. (2021) have similarly argued that high quality, critical journalism is essential for communicating and contextualizing research knowledge with public audiences. The authors view OS practices as both facilitating and complicating journalists’ work by providing a “wealth of available information” that would otherwise not be accessible. Finally, Arbuckle (2021) has highlighted that science journalists sometimes also provide material access to research, as they help bring findings that are not openly available to a wider public audience.

These OS-specific arguments echo broader conceptualizations of SJ as acting as a bridge between science and society that enables citizens to engage with research knowledge. For example, Ampollini and Bucchi (2020) argue that media coverage of research integrity issues could connect researchers with citizens, media, policy makers, and other research stakeholders in important discussions about the nature of science. More broadly, health and science journalists have been conceptualized as “brokers” of research knowledge ( Gesualdo *et al*., 2020; Pentzold *et al*., 2021; Yanovitzky & Weber, 2019) who can communicate, critique, and contextualize science and thus make it more “conceptually” accessible ( Kelly & Autry, 2013) and transparent in ways that are “societally-relevant” ( Elliott & Resnik, 2019). Applied to the OS context, the knowledge broker framework ( Yanovitzky & Weber, 2019) suggests that journalists have the potential to facilitate broader engagement with open research outputs by: 1) fostering public *awareness* of the OS and OA movements, 2) rendering open outputs (conceptually) *accessible* to nonacademic audiences, 3) *engaging* a wider public with debates around openness that are taking place within academia, 4) *linking* those debates to social issues or policies with public relevance, and 5) *mobilizing* open research findings to hold those in power to account when policies or decisions do not align with the available evidence. Such brokerage functions may enable journalists to build trust in science, as providing clear and understandable descriptions of OS practices involved in research can boost public credibility judgments of the findings ( Song *et al*., 2022).

Similarly, although health and science journalists fulfill some traditional journalistic roles—such as *watchdog* (holding powerful scientific or pharmaceutical institutions to account) and *agenda setter* (driving attention to new trends, issues, and findings in research)—they also play additional roles such as the *civic educator,* using their skills to teach audiences about the nature of scientific research and its limits and risks ( Fahy & Nisbet, 2011). These roles and functions, while not always consistently performed in practice, are important for ensuring that the growing trend towards openness in science supports the interests of society and the integrity of the scholarly record. For example, science journalists have acted as watchdogs by publishing nuanced, critical coverage of recent OS-related controversies, such as the use of predatory practices among major OA publishers ( Brainard, 2023; Kolata, 2017), flawed preprint studies ( Miller, 2022; Bartlett, 2023), and the high cost of article processing charges associated with OA publishing ( Ansede, 2023). Similarly, Retraction Watch—a blog and database founded and managed by science and health journalists—maintains a running list of retracted COVID-19 articles, including OA articles and preprints, and regularly features news about problematic research practices, including fraud, plagiarism, and predatory publishing in both closed and open science.

Science journalists’ ability to call attention to pernicious aspects of OS, while simultaneously helping publics take advantage of its benefits, makes them ideally positioned to help make scientific knowledge openly available, accessible and reusable for everyone, to increase scientific collaborations and sharing of information for the benefits of science and society, and to open the processes of scientific knowledge creation, evaluation, and communication to societal actors beyond the traditional scientific community ( UNESCO, 2021, p. 6).

That is, science journalists are ideally positioned to contribute to the “science communication” pillar of OS proposed in the influential UNESCO recommendations by brokering open research knowledge to public audiences. However, although scholars have highlighted this *potential* for journalists to contribute to the OS movement, very few studies have empirically examined journalists’ perceptions or use of open research outputs.

## Journalists’ pre-pandemic use of Open Access publications and preprints

### Pre-pandemic use of OA publications

Journalists have often been accused of “uncritically accepting sources’ designation of what is important and worthy of notice” ( Dunwoody, 2021, p. 20). This tendency—identified in journalists working across multiple beats—is likely to be more common among those who cover research-heavy topics, such as science and health, for two reasons. First, the complex, jargon-laden, and hyper-specialized nature of scientific work ( Baram-Tsabari *et al*., 2020; Ordway, 2022) means that journalists often rely heavily on the judgements of the scientists they interview to critique, contextualize, and verify new research findings ( Conrad, 1999; Hansen, 1994; Sebbah *et al*., 2022). Second, the mutual dependence of journalists on scientists (i.e., as sources of evidence and information) and scientists on journalists (i.e., as sources of public exposure and support) can encourage these groups to adopt one another’s norms and values ( Moorhead *et al*., 2022)—a phenomenon known as the *medialization of science* ( Peters *et al*., 2008; Weingart, 2012). Of course, tensions between journalistic and scientific values do arise ( Sponholz, 2010; Wihbey & Ward, 2016) and the impact of medialization may be more limited than previously theorized ( Lehmkuhl *et al*., 2023). Yet, medialization’s influence can be seen in media coverage of scholarly communications topics, such as peer review or research integrity, which mirror academic discourses and primarily present perspectives of scientists and scientific institutions ( Ampollini & Bucchi, 2020). While we found no English-language research investigating media coverage of the OA movement, it is likely that a similar trend exists.

Journalists’ internalization of scientific values may also influence how, or even whether, they use OA publications. What journalists consider ‘credible’ or ‘newsworthy’ often hinges on the perceptions of the scientists they interview ( Dunwoody, 2021). This may be one reason why some journalists preferentially cover research published in journals that are viewed as ‘prestigious’ or ‘reputable’ in the eyes of the academy, such as *Nature, Science, JAMA,* or *Proceedings for the National Academy of Science* ( Dumas-Mallet *et al*., 2017; Hansen, 1994; Lehmkuhl & Promies, 2020; MacLaughlin *et al*., 2018; Moorhead *et al*., 2021; Olvera-Lobo & Lopez, 2015; Rosen *et al*., 2016; Schäfer, 2011; St Lewis, 2011). The influence of journal reputation (itself often conflated with a journal’s Impact Factor; Morales *et al*., 2021) on journalists’ selection practices is so strong that it has been proposed as a core aspect of the science-specific news value of *scientific relevance,* reflecting the “Importance of an event for the scientific progress” ( Badenschier & Wormer, 2012, p. 73). Many of these journals have traditionally been closed access and now operate under a hybrid OA model (i.e., researchers can choose to publish their work OA for a fee).

Importantly, these high-impact journals also tend to have more resources to invest in science public relations (PR) efforts than other journals, enabling them to publish press releases and other press materials, circulate newsletters, and reach out to journalists to encourage them to cover newly released studies ( Nelkin, 1995). PR materials such as press releases have been termed “information subsidies” ( Granado, 2011) because they offer journalists the quotes, information, and context needed to craft science news stories with minimal time and effort. These same journals have also invested heavily in science news agencies, such as EurekAlert! and AlphaGalileo, which notify thousands of journalists worldwide about soon-to-be published research. These notifications provide journalists with early access to research under the condition that they adhere to an embargo (i.e., hold off on any media coverage until after a set date). Given growing demands on journalists’ time ( Massarani *et al*., 2021a), it is no surprise that PR efforts are consistently associated with increased coverage ( Comfort *et al*., 2022; Lehmkuhl & Promies, 2020; MacLaughlin *et al*., 2018). Science journalists’ heavy reliance on these information subsidies is thus an additional factor encouraging coverage of top, historically closed-access journals. It also encourages journalists to prioritize English-language, international research, rather than studies that may be more locally relevant ( Granado, 2011).

In addition, some US journalists report considering the Impact Factor of the journal when deciding which studies to cover ( Rosen *et al*., 2016; Schultz, 2023). Indeed, both the percentage of studies that receive news coverage and the number of news stories that are published per study tend to increase with the Impact Factor of the journal they were published in ( Dumas-Mallet *et al*., 2017). Although relying on heuristics like the Impact Factor may be a pragmatic practice for busy journalists, the concept of scientific relevance on which this metric is based is problematic for several reasons. First, the Impact Factor of a journal is not a valid marker of an individual paper’s quality and significance, although it is often used as one (e.g., within faculty review, promotion, and tenure decisions; McKiernan *et al.*, 2019). In addition, the metric tends to privilege research produced in English in the Global North (especially the US and UK) and published in major international journals ( Granado, 2011; Olvera-Lobo & Lopez, 2015), resulting in a lack of coverage of locally relevant research in the Global South ( Nguyen & Tran, 2019). It also does not bode well for some OA journals, many of which do not (yet) have an Impact Factor because they are not indexed in Clarivate’s Web of Science database ( Bergan, 2020) or, as newer journals, may not yet be established as ‘reputable’ sources in the eyes of scientists or the journalists who report on their work. Of course, these same reservations may also apply to some closed access journals, and may not be relevant to major OA journals with high Impact Factors and recognized brands, such as *PLOS Medicine* or *Nature Communications*. Still, exploratory research suggests that some journalists are “more suspicious of open access journals, believing they lacked a credible review process” ( Van Witsen & Takahashi, 2021, p. 10).

At the same time, journalists report that journal paywalls are a major barrier preventing their use of research ( Arbuckle, 2021; Boss *et al*., 2022; Gesualdo *et al*., 2020; Hinnant *et al*., 2017; Ordway, 2022), which may motivate them to rely on OA publications instead. This hypothesis is partially supported by existing evidence. Some studies suggest that OA publications receive more news coverage, on average, than their non-OA counterparts (e.g., Taylor, 2020; Wang *et al.*, 2015; Torres-Salinas *et al.*, 2020), while others find no evidence of such an “altmetric attention advantage” in news coverage (e.g., Alhoori *et al*., 2015). These seemingly conflicting findings may, in part, be explained by the alternative strategies journalists have developed for accessing paywalled research articles, such as obtaining copies direct from authors ( De Dobbelaer *et al*., 2018; Schultz, 2023), using subscription databases to which their institutions have access ( Boss *et al*., 2022), and relying on free summaries or abstracts rather than complete papers ( Bray, 2019). In addition, some major publishers make their libraries available to journalists who are members of specialized organizations, such as the Association of Health Care Journalists and the National Association of Science Writers (both based in the US). However, these privileges are not universal. Associations based in other countries, such as RedeComCiência (Brazilian Network of Journalists and Science Communicators), do not have the same partnerships in place, exacerbating asymmetries between the Global North and South. Some journalists may also be temporarily granted access to paywalled research as part of journals’ publicity efforts through the embargo system, as evidenced by the positive correlation between the promotion of research articles via embargo emails and their subsequent media coverage ( Lemke *et al*., 2022). This advance warning is meant to provide the time needed to interview sources, do background research, and, in theory, provide more nuanced and thorough coverage of the research ( Oransky, 2013). In practice, however, embargoes enable journals to restrict the flow of scientific information and to control media coverage of science by signaling which studies should be covered, by whom, and when ( Kiernan, 2003; Oransky, 2022).

It is also possible that the *type* of OA plays a role in whether or not a research article is used by journalists. Specifically, Schultz (2021) found that journalists preferentially cover articles from subscription journals that have been made OA at the expense of the authors (i.e., hybrid OA) or have been deposited in a publicly accessible form in an institutional repository (i.e., green OA), rather than those published in fully open journals (i.e., gold or diamond OA). While more research is needed, it is possible that journalists avoid using gold and diamond OA because of their suspicion of OA journals but have no such qualms about covering open research articles that have been published in closed (and thus ‘reputable’) journals. Indeed, a recent survey study by Schultz (2023) found that, while science journalists are generally positive about OA, they are more willing to cite papers from hybrid rather than gold OA journals. However, as discussed above, it is also possible that hybrid and closed access journals have more resources to invest in publishing press releases and other forms of science PR and are thus more successful in garnering media coverage ( Lehmkuhl & Promies, 2020; MacLaughlin *et al*., 2018).

Finally, the ability to circumvent paywalls is not distributed equally among all journalists. Many of the access strategies discussed above—such as requesting articles from authors or using databases—tend to require time and resources that some journalists simply do not have. This is particularly likely for journalists based in the Global South ( Nguyen & Tran, 2019), those working for digital, rather than print, publications ( Manninen, 2017), those without subject-specific training ( Leask *et al*., 2010), and journalists with less advanced information literacy skills, such as students or inexperienced reporters ( Boss *et al*., 2022).

### Pre-pandemic use of preprints

While journal reputation, science PR, and access barriers are important factors in journalists’ engagement with OA publications, their use of preprints is strongly connected to perceptions and beliefs about peer review. Research suggests that journalistic discourses surrounding peer review tend to mirror those found in academic debates, portraying peer review as a “guarantee of good science” and the “cornerstone of maintaining the quality” of research ( Ampollini & Bucchi, 2020, p. 466; Sebbah *et al*., 2022). As such, many journalists may be weary of OS initiatives that challenge traditional notions of peer review, such as preprints. For example, Dunwoody (2021) argues that journalists’ reliance on interviews with scientific experts means that those experts can “easily sell the argument that journalists must respect the scientific process and, for example, must wait for peer review to take place before embarking on a wider dissemination of research results” (p. 20; also, Oransky, 2022). Indeed, many science journalists “assume that peer review assures quality control of the science” ( Conrad, 1999, p. 286; also Forsyth *et al*., 2012) and professional journalism organizations have been known to discourage the use of unreviewed science ( Associated Press, 2020; Fox, 2018). This is particularly true for controversial topics that are newsworthy—that is, those issues that have the potential to generate the most misinformation or confusion among the public ( Science Media Centre, n.d.).

Many of these controversial, newsworthy research topics are found in the life sciences, an umbrella term encompassing many health- and medicine-related research fields. These fields are unique in their historically low levels of preprint use ( Puebla *et al*., 2022), high levels of press release promotion ( Lemke *et al*., 2021; Orduña Malea & Costas, 2023), and correspondingly large volumes of media coverage ( Banshal *et al*., 2019; Ginosar *et al*., 2022; Joubert *et al*., 2022). With potential to directly influence health policy, medical practice, and public wellbeing, the risks associated with posting and promoting preprints are also arguably greater in health-related fields than in other research areas ( Bonnechère, 2020; Chung, 2020; Maslove, 2018), raising additional concern about the use of health-related preprints in journalism. UK’s Science Media Centre Director Fiona Fox (2018) emphasized these risks in an open letter on her blog titled “the preprint dilemma: good for science, bad for the public?” In it, she urged scholars, academic publishers, and science communicators to consider the wider impacts of preprint use, particularly within the controversial, newsworthy research areas on which the SMC focuses.

Many of Fox’s concerns—and those of the scholars who would come after her—centered on the ways in which preprints can disrupt the system of “checks and balances” that she saw as essential for supporting accurate, trustworthy science media coverage. This system, which is still largely in place today, relies heavily on the peer review process as a quality control mechanism and embargo system as a source of story ideas (as discussed above). While embargoes are controversial ( Altman, 1996; Oransky, 2013), Fox (2018) argued that they offer journalists the time needed to more thoroughly vet and communicate the research they cover—time they would otherwise not have in a “24-hour rolling news” cycle that privileges newness and originality over accuracy and rigor. In a world with preprints as news sources, Fox (2018) feared that embargoes would no longer be possible—and that the resulting damage would be irreparable. “The critical point is this,” she wrote, “once these findings have been reported in one or two national newspapers they cannot be unreported.”

Fox’s letter was quickly followed by an opinion piece in *Nature*, in which SMC senior press manager Tom Sheldon (2018a) amplified Fox’s concerns to more than 3 million online monthly readers ( “Announcement: A new iPad app for Nature readers,” 2012; also Sheldon, 2018b). This pivotal moment brought fears about preprint coverage into the mainstream scholarly discourse, but also sparked some of the first arguments in defense of preprint-based news coverage. In a series of comments responding to Sheldon’s (2018a) article, scholars and OS advocates highlighted the limitations of relying on peer review as a quality control mechanism ( Tennant *et al*., 2018), arguing that media coverage of preprints and peer-reviewed articles posed similar risks to public wellbeing ( Sarabipour, 2018). Underpinning the responses to Sheldon’s piece was a belief that “the tension between supporting preprints and good journalism is a false dichotomy” ( Sarabipour, 2018); that the benefits of preprints for science outweighed any potential risks for the public ( Sarabipour, 2018; Sarabipour *et al*., 2018); and that, rather than suppressing preprint-based journalism, scholars and journalists could work together to support accurate and engaging science media coverage ( Fraser & Polka, 2018; Sarabipour *et al*., 2018).

The body of scholarship summarized above advanced important arguments about the potential risks and benefits of preprint-based media coverage and provided some of the first anecdotal evidence that journalists occasionally covered preprints before the pandemic. For example, Sheldon (2018a) reported that journalists had started “trawling” preprint servers for potential story ideas and argued that this practice had the potential to put news audiences at risk. Similarly, Sarabipour (2018) argued that “Responsible journalists already report on preprints with the help of real-time commentary from scientists on Twitter and elsewhere,” citing a story in *The Atlantic* by journalist Ed Yong (2016) that featured tweets about a bioRxiv preprint by Sender *et al*. (2016) as an example. Molldrem *et al*. (2021) have also noted that arXiv preprints have at least occasionally been (mis)used by journalists before the pandemic, as evidenced by widespread coverage of a problematic study of cold fusion posted to the server in 2013. While each of these examples is anecdotal on its own, collectively they provide preliminary evidence that at least some journalists occasionally covered preprints before the pandemic, and that social media may have helped them to do so.

## Journalists’ use of Open Access publications and preprints during the COVID-19 pandemic

### Pandemic use of OA publications

Surprisingly, we found almost no research examining journalists’ engagement with OA publications during the pandemic. One exception is a survey study of US-based science journalists examining how COVID-19 had changed their knowledge or perceptions of OA, which in this case was defined as including both OA publications and preprints ( Schultz, 2023). The study found that most journalists had been familiar with OA before the pandemic, although COVID-19 may have increased their knowledge of certain forms of OA, such as green OA. While this study provides some of the first insights into how journalists perceive the OA movement and how the pandemic has changed these perceptions, the generalizability of the findings is limited by the small and nonrandom nature of the sample. More research is needed to better understand whether or how the pandemic has shifted journalists’ perceptions of, and willingness to use, OA publications, particularly beyond the US context.

Similarly, our review of the literature suggested that scholars have yet to explicitly examine media coverage of OA versus closed access publications during the COVID-19 pandemic. Scholars have compared social media attention to open and closed access COVID-19 publications (e.g., Torres Salinas *et al*., 2020), as well as journalistic coverage of preprints (discussed in the next section). Yet, none to our knowledge have focused on articles published in OA journals or available through green OA. It is possible that the lack of research is due to the methodological and data quality-related challenges of tracking media coverage of research ( Fleerackers *et al*., 2022), as well as disciplinary norms for studying science journalism. With a few exceptions ( Matthias *et al*., 2020; Van Schalkwyk & Dudek, 2022), SJ and communication scholars tend to identify science news stories using topic-related keyword searches, rather than by searching for coverage of specific research outputs ( Fleerackers *et al*., 2022; Hansen, 2009). It is also possible that the lack of interest in this topic is linked to the fact that almost all COVID-19 research was made OA during the early pandemic period, even if only temporarily ( Besançon *et al*., 2021; Engebretson, 2020). We discuss the urgent need for more studies in our Recommendations for future work.

### Pandemic use of preprints

The onset of the COVID-19 pandemic delivered exactly the type of widespread coverage of preprints in controversial, health-related fields that Fox and Sheldon feared, bringing new urgency to what had been a mostly theoretical debate back in 2018 ( Molldrem *et al*., 2021). The early months of the crisis saw a sharp increase in the volume of available COVID-19-related preprints ( Else, 2020; Horbach, 2020; Watson, 2022) and an “Increased permeability between scholarly circles, the news media, and the lay public” ( Molldrem *et al*., 2021, p. 1470), with preprint servers such as medRxiv and bioRxiv becoming key disseminators of pandemic research ( Vergoulis *et al*., 2021). Given the lack of peer-reviewed evidence about the virus available at the time, COVID-19-related preprints became a key source of information for journalists ( Fraser *et al*., 2021; Majumder & Mandl, 2020). While much of the resulting media coverage was helpful or benign, flawed and controversial preprints also made headlines (see Majumder & Mandl, 2020; Molldrem *et al*., 2021; Scheirer, 2020; van Schalkwyk *et al*., 2020, for reviews of these cases). Concerns about misinformation—similar to those discussed back in 2018—resurfaced, with scholars arguing that “conversations surrounding individual non-peer-reviewed preprints has made it difficult to extract meaningful signals about reliable, cumulative scientific evidence from the noise of sometimes short-lived findings” [sic] ( Brossard & Scheufele, 2022, p. 614) and warning that “uncontrolled and potentially misleading information will reach the general public, directly or via the media, leading to incorrect, sometimes fatal, responses to the pandemic” ( Chirico *et al*., 2020, p. 300).

Despite these fears, COVID-19-related preprints appear to have stood up relatively well to the scrutiny of peer review ( Kodvanj *et al*., 2022; Nelson *et al*., 2022; Otridge *et al*., 2022; Zeraatkar *et al*., 2022), although a minority do appear to have changed in important ways between initial posting and journal publication ( Brierley *et al*., 2022) or been retracted ( Abritis *et al*., 2021; Santos-d’Amorim *et al*., 2021). Scholars have proposed that the use of OS practices such as open data could help prevent misleading coverage of preprint research and improve the quality of SJ overall ( Breznau *et al*., 2020). Others have argued that journalism could similarly mitigate the potential risk of misinformation by identifying and providing early, critical coverage of the preprints that are most likely to cause considerable damage to the public ( Stollorz, 2021). This dual role of journalism—as both a cause and antidote for the spread of preprint-based misinformation—aligns with recent proposals that communicating OS outputs to public audiences can be both enriching (i.e., if it improves public perceptions, awareness, and knowledge of science) and misleading (i.e., if research outputs are not communicated with care) ( Ho *et al*., 2021; Vignoli & Rörden, 2019).

Some evidence suggests that news coverage of COVID-19-related preprints outstripped preprints on other subjects, at least during the early months of the pandemic. In the US, UK, Brazil, Germany, and South Africa, journalists from diverse media outlets drew on COVID-19-related preprints as sources of coverage ( Fleerackers *et al*., 2022; Massarani *et al.*, 2021a; Massarani & Neves, 2022; Simons & Schniedermann, 2023; Van Schalkwyk & Dudek, 2022). A widely cited study by Fraser *et al*. (2021) found that more than a quarter of COVID-19-related preprints posted to bioRxiv and medRxiv during the first ten months of COVID-19 were mentioned in at least one media story, while only about 1% of those on other topics received media coverage. Besançon *et al.* (2021) found that COVID-19-related preprints posted to arXiv, medRxiv, and bioRxiv between January and July 2020 each received more coverage in blogs and news stories than non-COVID-19-related preprints posted to arXiv during the same time period. Similarly, coverage of preprints in German news outlets was relatively low before the pandemic, but surged in 2020 and 2021 ( Simons & Schniedermann, 2023). Finally, a study found that preprints were featured in less than 2% of media coverage of research before the pandemic, but that this proportion surged to almost 4% after the onset of COVID-19 ( Fleerackers *et al.*, 2023). Moreover, this surge appeared to be driven entirely by COVID-19 preprints, as the launch of the medical preprint server medRxiv in 2019 had little or no effect on rates of preprint coverage. Some journalists describe this widespread adoption of preprints as a “paradigm shift” that is likely to persist post-pandemic ( Fleerackers *et al*., 2022). Scholars have made similar claims that the recent coverage of preprints represents a long-term “cultural shift” in journalism ( Fraser *et al*., 2021, p. 18; Stollorz, 2021; Van Schalkwyk & Dudek, 2022).

However, other studies have found that preprints were less influential within COVID-19 journalism than the dominant discourse suggests. For example, a small study found no significant difference in the amount of media coverage received by medRxiv preprints and peer-reviewed publications about COVID-19-related therapies that were posted between February 1 and May 10, 2020 ( Jung *et al*., 2021). Kousha and Thelwall (2020) found that the five COVID-19-related research articles that received the most media coverage were all peer-reviewed publications. Similarly, journalists from around the world have reported that they drew primarily on peer-reviewed publications and interviews with local scientists for their pandemic coverage, with preprints acting as a more secondary information source ( Massarani *et al*., 2021b). This finding is supported by comments from some of the journalists interviewed by Fleerackers *et al*. (2022), who claimed that they “doubt[ed] that arXiv is the place a lot of medical reporters are going to eagerly pull reporting from” (p. 11) post-pandemic. In addition, although journalists feel positive about open research in general—even more now than before the pandemic—they remain more skeptical of preprints than OA journal publications ( Schultz, 2023). Moreover, it is possible that the volume of preprint-coverage varies across geographies, media outlets, and individual journalists. For example, Massarani *et al*. (2021a) found that journalists in the Asia/Pacific region were among the most likely to use preprints, whereas those in African and Middle Eastern countries were among the least likely. In addition, Fleerackers *et al.* (2023) found little or no change in the coverage of non-COVID-19 preprints during the pandemic period, suggesting that journalists’ embrace of COVID-19 preprints may not extend to preprints on other topics, nor those posted during less urgent crisis contexts.

Regardless of how the volume of preprint news coverage has changed as a result of COVID-19, preprint-based journalism seen during the pandemic appears to be qualitatively different from “normal” SJ ( Fleerackers *et al*., 2022). While transparency and accuracy are key tenets of ethical, high quality journalism ( Kovach & Rosenstiel, 2021; *SPJ Code of Ethics - Society of Professional Journalists*, n.d.), journalists do not consistently uphold these standards when covering preprints, with between 42-61% of preprint-based media stories failing to disclose the unreviewed nature of the preprints they reported ( Fleerackers *et al*., 2022; Oliveira *et al*., 2021; Van Schalkwyk & Dudek, 2022). A study of the German media landscape before and after the pandemic found similar results, with descriptions of preprints becoming more tentative during the pandemic—even for stories that were unrelated to COVID-19 ( Simons & Schniedermann, 2023). The lack of consistency in reporting can be problematic, given that “the framing of a reporter’s coverage … can sensationalize and distort preliminary findings, particularly when there is uncertainty, disagreement, and confusion among experts” ( Molldrem *et al*., 2021, p. 1476). To prevent such distortions, scholars have argued that journalists should adopt more standardized procedures for covering preprints, such as drawing on outside expertise to vet the results and labeling results as “under review” or “preprint research” ( Ginsparg, 2021; Dunwoody, as quoted in Hamilton, 2020). Interestingly, although many journalists reported adopting both of these novel practices to cover preprints during the pandemic ( Fleerackers *et al*., 2022; Massarani *et al*., 2021c; Schultz, 2023), they are also skeptical of the effectiveness of these measures. Specifically, journalists feel they lack the expertise (not to mention time) to verify preprint research and believe audience members are unlikely to know the term ‘preprint’ or understand how peer review works ( Fleerackers *et al*., 2022). While results are mixed, a growing body of research suggests that public understanding of preprints *is*, indeed, limited—at least in the US ( Ratcliff *et al*., 2023; Wingen *et al*., 2022).

## Recommendations for future work

In reviewing the literature discussed in the preceding sections, we have identified several gaps and directions for future research, which we outline below.

### Key gaps in research on journalists’ use of OA publications

Somewhat surprisingly, we have not been able to identify any studies that examine how and to what extent journalists have used OA publications during the COVID-19 pandemic. While a few studies have looked at journalists’ perceptions and use of pandemic-related preprints, other types of open research outputs—including but not limited to OA publications—have been largely overlooked in the research literature. More broadly, few studies so far have examined how journalists perceive the OA movement and its relevance to their work, how they view OA journals and articles, and whether the pandemic has changed these attitudes and to what extent. In addition, research is needed to understand whether engagement with OA research and exposure to the OS values associated with it might push science journalists to reflect on their own values, practices, roles, or norms. Very little is known about how journalists find and access closed access publications, and whether access barriers are greater for certain kinds of journalists, such as freelancers, generalists, and journalists based in the Global South.

### Key gaps in research on journalists’ use of preprints

It has been suggested that the COVID-19 pandemic led to a professional paradigm shift in terms of media coverage of preprints; however, we don’t have a clear sense of how often and for what purposes journalists covered preprints pre-pandemic. There is a particular need for studies examining journalists’ use of preprints before the COVID-19 outbreak and during other recent outbreaks (e.g., Ebola, Zika). Longitudinal research is also needed in order to highlight changes in preprint coverage over time, identify patterns and shifts in attitudes or behavior, and assess the impact of COVID-19 on journalistic practices and norms. Examining changes in journalists’ use of preprints beyond the pandemic is especially important as preprints, themselves, continue to evolve. For example, as more and more preprint review services come online ( Henriques *et al.*, 2023), future research could examine how journalists perceive and use preprints that have been peer-reviewed outside of the traditional journal publishing system.

In a similar vein, much has been written about the potential of preprints to elicit public confusion and misinformation, yet only a handful of case studies have examined the flow of misinformation from preprints to media and public discourse. How much preprint coverage actually contributed to pandemic misinformation remains unknown—which is crucial to understand in preparation for future public health crises. Evidence in this regard would also help inform the current debate on the benefits and pitfalls of preprints, which at this point remains largely speculative. More broadly, it is unclear how audiences understand and respond to the descriptions of preprints they encounter in the news and how journalists can best communicate the unreviewed nature of preprint knowledge without losing audience trust in science or in journalism. ( Ratcliff *et al.*, 2023).

### Gaps in global, intersectional research on OS-based journalism

Finally, our review suggests that research examining journalists’ use of open research outputs beyond the Global North is sorely needed. As Rao (2019) has identified, journalists and audiences in the Global South are uniquely affected by “gender, race, sexuality, caste, and various other forms of exclusions [that] play out in multiple arenas” (p. 702). Our understanding of OS-based journalism will remain incomplete unless we examine how such exclusions shape the nature of the news in these countries, which house the majority of the world’s population yet are so often overlooked in journalism scholarship ( Wright *et al*., 2019). As this literature review largely focused on English-language literature, conducting a review of contributions published in other languages would be an important first step towards filling this gap. For example, Brazilian initiatives such as SciELO and the Bori Agency have launched PR efforts to increase the public visibility of OA publications ( Packer, 2014; Righetti *et al*., 2022). In addition, discussions on how bridging OA and science communication could promote reflections on issues related to science, society, and democracy have gained strength in Brazil ( Barata, 2022). Yet, these initiatives and discourses have not been well-represented in international databases and metrics ( Barata, 2019).

More broadly, we lack research examining how journalists’ use of open research outputs depends on aspects of their identity and professional context (e.g., their gender, education, status as a freelancer/staff member, nature of the media outlet(s) they work for). Such research is needed given the increasing diversification and expansion of (science) journalism professionals, formats, and practices ( Ginosar *et al*., 2022; Schapals, 2022) and growing awareness that journalists’ experiences are not universal but rather shaped by the intersections of their identities, contexts, and backgrounds ( Jackson, 2022; Massarani *et al*., 2021a; Mesmer, 2022).

## Conclusion

Open science seeks to make science accessible to all, including non-experts, decision-makers, and the public at large. However, OS cannot fulfill its democratic potential “if those who are unfamiliar with the research world do not know how to seek […] openly available research, and have difficulty parsing the meaning once they do” ( Arbuckle, 2021, p. 103). Communicating open scientific findings and processes with everyone in an understandable and accessible language is, therefore, essential for increasing the societal impact of OS. For this reason, open science needs science journalism. Yet, despite the potential for SJ to contribute to the OS movement by making open research knowledge more conceptually accessible, little is known about journalists’ use of open outputs or adherence to OS values. Through a narrative synthesis of the scant scholarship that has examined the intersection of OS and SJ, this review simultaneously took a first step towards filling this gap and revealed the many additional questions that remain unanswered. As OA publications, preprints, and other forms of OS become increasingly mainstream among researchers, addressing these known unknowns is essential: for scientists, journalists, and the publics they serve.

## Data Availability

No data are associated with this article.
