# Supplementary material for: Making science public: a review of journalists’ use of Open Access research
Source: F1000Res. 2024 Jan 2;12:512. Originally published 2023 May 18. [Version 2] doi: 10.12688/f1000research.133710.2 (PMC10618641; doi:10.12688/f1000research.133710.2)
Supplement: Peer review history

# Reviewer report: Reviewer response for version 2

**DOI:** 10.5256/f1000research.159997.r234926

**Published:** 2024-02-05

**Authors:** Alyssa Arbuckle

**Affiliations:** 1 University of Victoria, Victoria, British Columbia, Canada

A thoughtful and clarifying revision of an already compelling paper.

Is the review written in accessible language?

Yes

Are all factual statements correct and adequately supported by citations?

Yes

Are the conclusions drawn appropriate in the context of the current research literature?

Yes

Is the topic of the review discussed comprehensively in the context of the current literature?

Yes

Reviewer Expertise:

Open access, open scholarship, scholarly communication

I confirm that I have read this submission and believe that I have an appropriate level of expertise to confirm that it is of an acceptable scientific standard.

# Reviewer report: Reviewer response for version 2

**DOI:** 10.5256/f1000research.159997.r234928

**Published:** 2024-01-24

**Authors:** Ivan Oransky

**Affiliations:** 1 The Center For Scientific Integrity, Retraction Watch, New York, NY, USA; 2 Journalism, New York University, New York, New York, USA

No additional comments

Is the review written in accessible language?

Yes

Are all factual statements correct and adequately supported by citations?

Yes

Are the conclusions drawn appropriate in the context of the current research literature?

Yes

Is the topic of the review discussed comprehensively in the context of the current literature?

Yes

Reviewer Expertise:

Science and medical journalism, research integrity, publishing

I confirm that I have read this submission and believe that I have an appropriate level of expertise to confirm that it is of an acceptable scientific standard.

# Reviewer report: Reviewer response for version 1

**Published:** 2023-11-08

**Authors:** Henrik Karlstrøm

**Affiliations:** 1 Nordic Institute for Studies of Innovation, Research and Education, Oslo, Norway

Given that increased public engagement with scientific research is an impetus of the Open Science movement itself, the research question of this paper is highly relevant. The authors do a good job of establishing the ways in which brokerage between science and journalism can improve public understanding of the scientific process. I find the paper well-structured, well-argued and discussing a topic of potentially high import.

 The paper is quite limited in scope. The authors note that there are few empirical investigations of science journalists' engagement with OS literature, and state that "this review mainly covers research and theoretical contributions that discuss the intersections of OS and SJ tangentially or as a secondary concern, rather than a primary focus". It is good that the authors acknowledge the limitations of reviewing a literature that is only tangentially concerned with the question at hand. You can only work with what is available, of course, and the authors do note that an empirical investigation of journalists' use of code and data is planned in the future. However, this limitation to conceptual discussions obviously affects the scope of the findings claimed by the paper.

 Given the lack of empirical data, the authors are correct to call this a review of existing literature. There is a discrepancy between how this is framed in the abstract and in the key recommendations. The abstract states: "We find that, despite journalists’ potential to act as critical brokers of open access knowledge, their use of open research outputs is hampered by an overreliance on traditional criteria for evaluatingscientific quality; concerns about the trustworthiness of open research outputs; and challenges using and verifying the findings." However, these are not really findings, given that the literature reviewed does not directly address these questions. A better framing would have been to focus on the gaps identified in the literature, as these are amply demonstrated in the paper.

 Overall, this is a useful overview of the literature that exists at the crossroads of science journalism, open science and COVID research. It constitutes a taking stock of the current situation and a roadmap for further investigation into the factors that shape decisions by non-scientists who are trying to navigate the somewhat insular world of academic publishing.

Is the review written in accessible language?

Yes

Are all factual statements correct and adequately supported by citations?

Yes

Are the conclusions drawn appropriate in the context of the current research literature?

Yes

Is the topic of the review discussed comprehensively in the context of the current literature?

Yes

Reviewer Expertise:

Scientific publishing, metascience, bibliometrics

I confirm that I have read this submission and believe that I have an appropriate level of expertise to confirm that it is of an acceptable scientific standard.

# Response

**Published:** 2023-12-13

**Authors:** Natascha Chtena

**Affiliations:** Scholarly Communications Lab, Simon Fraser University, Vancouver, British Columbia, Canada

Thank you for taking the time to read our paper and for providing valuable comments for its improvement. To streamline the abstract with our recommendations for future work, we have revised the abstract to further highlight the gaps identified in the literature:  

*Science journalists are uniquely positioned to increase the societal impact of open research outputs by contextualizing and communicating findings in ways that highlight their relevance for non-specialist audiences. Yet, it is unclear to what degree journalists use open research outputs, such as open access publications and preprints, in their reporting; what factors motivate or constrain this use; and how the recent surge in openly available research seen during the COVID-19 pandemic has affected this. This article examines these questions through a review of relevant literature published from 2018 onwards, particularly literature relating to the COVID-19 pandemic. We find that research that explicitly examines journalists’ engagement with open access publications or preprints is scarce, with existing literature mostly addressing the topic tangentially or as a secondary concern, rather than a primary focus. Still, the limited body of evidence points to several factors that may hamper journalists’ use of these outputs and thus warrant further exploration. These include an overreliance on traditional criteria for evaluating scientific quality; concerns about the trustworthiness of open research outputs; and challenges using and verifying the findings. We also find that, while the COVID-19 pandemic encouraged journalists to explore open research outputs such as preprints, the extent to which these explorations will become established journalistic practices remains unclear. Furthermore, we note that current research is overwhelmingly authored and focused on the Global North, and the United States specifically. We conclude with recommendations for future research that attend to issues of equity and diversity, and more explicitly examine the intersections of open access and science journalism.*

# Reviewer report: Reviewer response for version 1

**DOI:** 10.5256/f1000research.146721.r214232

**Published:** 2023-10-31

**Authors:** Moumita Koley

**Affiliations:** 1 Indian Institute of Science Bangalore, Bangalore, Karnataka, India

This is a timely and relevant article, especially since open science is an essential discussion in the scholarly community. The UNESCO Open Science recommendations highlight the importance of the accessibility of science to the broader public. As mentioned in the article, it is, however, not always possible for the general public to understand highly specialized scientific articles, and science journalism can be really beneficial in bringing complex knowledge in a simpler form to people. In this regard, the accessibility of scholarly articles to journalists is an important consideration and enabler as well. So, how open access is enabling better science journalism needs to be understood. However, as a scholar who studies open science from a different perspective, my knowledge of science journalism and related literature is not adequate to evaluate the quality of the literature review provided here. Also, I assume that this article is a review of literature addressing science journalism and open science, not "a review of a review of...".

 This article presents a review of literature that mainly addresses the questions of open access rather than the broader concept of open science. Open science is a much broader concept where open access is an element. So unless the literature addresses the use of other elements, such as open data, code, etc., by the science journalists, in a more accurate sense, this article tracks mostly the use of open-access research by journalists. Using open science and open access interchangeably does not help the cause of open science as a broad concept.

 Since 'Altmetrics' is becoming increasingly popular in assessing the impact of research, especially on society, reporting any study by journalists' impact on the altmetric scores will be an interesting observation. 

 The method and value of science is deeply rooted in the peer-review process. As indicated by the cold fusion reporting through preprint and the recent controversy of widespread coverage of the LK-99 as the potential superconductor (not mentioned in the present article), indicates the problematic nature of covering preprint by SJs. Also, how the newer trend of peer-reviewed preprints are used by the SJs and how these newer model of publications can be used to remove confusion regarding the quality of preprints could be an exciting addition to this article.

Is the review written in accessible language?

Yes

Are all factual statements correct and adequately supported by citations?

Yes

Are the conclusions drawn appropriate in the context of the current research literature?

Partly

Is the topic of the review discussed comprehensively in the context of the current literature?

Yes

Reviewer Expertise:

Open science, research evaluation, open access

I confirm that I have read this submission and believe that I have an appropriate level of expertise to confirm that it is of an acceptable scientific standard.

# Response

**Published:** 2023-12-13

**Authors:** Natascha Chtena

**Affiliations:** Scholarly Communications Lab, Simon Fraser University, Vancouver, British Columbia, Canada

Thank you for taking the time to read our paper and for providing valuable comments for its improvement.

**Comment 1: This article presents a review of literature that mainly addresses the questions of open access rather than the broader concept of open science. Open science is a much broader concept where open access is an element. So unless the literature addresses the use of other elements, such as open data, code, etc., by the science journalists, in a more accurate sense, this article tracks mostly the use of open-access research by journalists. Using open science and open access interchangeably does not help the cause of open science as a broad concept.** 

 To clarify our focus on OA journal publications and preprints, we have changed the title to: *Making science public: A review of journalists’ use of open access research*

 In addition, we have reframed the abstract to focus more specifically on OA journal articles and preprints, not OS broadly defined. At the same time, we feel it is important to contextualize journalists’ engagement with OA and preprints within the wider OS movement, given that OA/preprints are a part of OS and should be considered with the wider motivations and goals of openness in mind. We have taken care not to use the terms OS and OA interchangeably, referring to OA pubs and preprints as ‘open research outputs’ and OS when discussing the movement as a whole. 

**Comment 2: Since 'Altmetrics' is becoming increasingly popular in assessing the impact of research, especially on society, reporting any study by journalists' impact on the altmetric scores will be an interesting observation.**

 Altmetrics continue to be displayed on journal websites, but we are not aware of evidence that suggests that they are being used for research assessment. However, we agree that journalism can affect the altmetric scores of a paper, which appears to be tied to the type of access journalists have to the work. We mentioned this in our original manuscript but have provided additional references to strengthen the claim as follows:

*Some studies suggest that OA publications receive more news coverage, on average, than their non-OA counterparts (e.g., Taylor, 2020; **Wang et al., 2015; Torres-Salinas et al., 2020**), while others find no evidence of such an “altmetric attention advantage” in news coverage (e.g., Alhoori et al., 2015).*

 In addition, the last two sentences of the paragraph link access to embargoed copied with higher media coverage, which would also lead to higher citations. 

**Comment 3: The method and value of science is deeply rooted in the peer-review process. As indicated by the cold fusion reporting through preprint and the recent controversy of widespread coverage of the LK-99 as the potential superconductor (not mentioned in the present article), indicates the problematic nature of covering preprint by SJs. Also, how the newer trend of peer-reviewed preprints are used by the SJs and how these newer model of publications can be used to remove confusion regarding the quality of preprints could be an exciting addition to this article.**

 We agree that journalists’ perception and use of peer-reviewed preprints is an exciting avenue for future research and have noted this in our recommendations for future work:

*Longitudinal research is also needed in order to highlight changes in preprint coverage over time, identify patterns and shifts in attitudes or behavior, and assess the impact of COVID-19 on journalistic practices and norms. **Examining changes in journalists’ use of preprints beyond the pandemic is especially important as preprints, themselves, continue to evolve. For example, as more and more preprint review services come online (Henriques et al., 2023), future research could examine how journalists perceive and use preprints that have been peer reviewed outside of the traditional journal publishing system.***

# Reviewer report: Reviewer response for version 1

**DOI:** 10.5256/f1000research.146721.r214228

**Published:** 2023-10-31

**Authors:** Alyssa Arbuckle

**Affiliations:** 1 University of Victoria, Victoria, British Columbia, Canada

This article examines the intersection of open scholarship and science journalism. The authors suggest that this is an understudied area, despite the critical importance of open access research to journalism and vice versa. They provide a thorough literature review of the research that is available on science journalists’ use of open access research. Notably, the authors dig into the actual usage of preprints by science journalists during the COVID-19 pandemic, which had become a hot button topic for those working in the field.

 I would be interested to see one of the arguments of this article fleshed out slightly; that is, for the authors to interrogate the value of specialist language more deeply. It is a truism of academic research that it is obscure and opaque to anyone outside a specific discipline (and sometimes sub-discipline). Indeed, this is one of the areas where science journalists and other knowledge brokers come into play: as translators for a broader, more generalized readership. But it is important, too, to not lose sight of the value of shorthand. As Laura Mandell writes in Breaking the Book: Print Humanities in the Digital Age, shorthand allows for an expedited exchange of ideas between specialists without having to include all of the detailed context and history of each theory or concept being explored and built upon. Translation and comprehension are necessary, of course; but there is value to the complexity and detail of research, too. How does this tension affect the production, circulation, and uptake of open access research?

 I also found the point about science journalists relying on prestige markers such as the Impact Factor an angle that would be well worth digging more deeply into. Prestige and open access publishing are often critiqued within the context of hiring, review, or tenure and promotion. What does it mean that science journalists and hiring and promotion committees are depending on the same arbitrary and easily gamed metrics?

 Overall, this article provides an important summation of the current state of play regarding science journalism and open scholarship. It highlights that for open access research to reach its full impact, it must be findable, legible, and reusable; science journalism is one of the mechanisms to reach such a goal. The article closes with recommendations for future work, all of which would be well worth pursuing. I will look forward to reading more from the lead author and her co-authors on the subject.

Is the review written in accessible language?

Yes

Are all factual statements correct and adequately supported by citations?

Yes

Are the conclusions drawn appropriate in the context of the current research literature?

Yes

Is the topic of the review discussed comprehensively in the context of the current literature?

Yes

Reviewer Expertise:

Open access, open scholarship, scholarly communication

I confirm that I have read this submission and believe that I have an appropriate level of expertise to confirm that it is of an acceptable scientific standard.

# Response

**Published:** 2023-12-13

**Authors:** Natascha Chtena

**Affiliations:** Scholarly Communications Lab, Simon Fraser University, Vancouver, British Columbia, Canada

Thank you for taking the time to read our paper and for providing valuable comments for its improvement. 

**Comment 1: I would be interested to see one of the arguments of this article fleshed out slightly; that is, for the authors to interrogate the value of specialist language more deeply. It is a truism of academic research that it is obscure and opaque to anyone outside a specific discipline (and sometimes sub-discipline). Indeed, this is one of the areas where science journalists and other knowledge brokers come into play: as translators for a broader, more generalized readership. But it is important, too, to not lose sight of the value of shorthand. As Laura Mandell writes in Breaking the Book: Print Humanities in the Digital Age, shorthand allows for an expedited exchange of ideas between specialists without having to include all of the detailed context and history of each theory or concept being explored and built upon. Translation and comprehension are necessary, of course; but there is value to the complexity and detail of research, too. How does this tension affect the production, circulation, and uptake of open access research?**

 Thank you for this suggestion. ****We have revised the second paragraph of the Introduction to highlight the value and utility of specialist language when used among experts: 

*Academic publications are written for peer researchers and academics rather than the general public and use the jargon, rhetorical features, and communication norms and conventions of the disciplines within which they are produced ( Fahnestock, 1986). **Such specialist language can enhance understanding within these disciplinary communities, contributing to more economical, precise communication that supports collaboration among experts (Hirst, 2003). However,** it can be very difficult for ‘lay’ readers to understand.*

 Hirst, R. (2003). Scientific jargon, good and bad. Journal of Technical Writing and Communication, 33(3), 201–229. https://doi.org/10.2190/J8JJ-4YD0-4R00-G5N0

**Comment 2: I also found the point about science journalists relying on prestige markers such as the Impact Factor an angle that would be well worth digging more deeply into. Prestige and open access publishing are often critiqued within the context of hiring, review, or tenure and promotion. What does it mean that science journalists and hiring and promotion committees are depending on the same arbitrary and easily gamed metrics?**

 We have added a sentence noting the problematic use of the IF within RPT processes: 

*Although relying on heuristics like the Impact Factor may be a pragmatic practice for busy journalists, the concept of scientific relevance on which they are based is problematic for several reasons. **First, the Impact Factor of a journal is not a valid marker of an individual paper’s quality and significance, although it is often used as one (e.g., within faculty review, promotion, and tenure decisions; McKiernan et al., 2019 ). **In addition, the metric also tends to privilege research produced in English in the Global North (especially the US and UK) and published in major international journals ( Granado, 2011; Olvera-Lobo & Lopez, 2015) resulting in a lack of coverage of locally relevant research in the Global South ( Nguyen & Tran, 2019).*

# Reviewer report: Reviewer response for version 1

**DOI:** 10.5256/f1000research.146721.r201145

**Published:** 2023-10-31

**Authors:** Ivan Oransky

**Affiliations:** 1 The Center For Scientific Integrity, Retraction Watch, New York, NY, USA; 2 Journalism, New York University, New York, New York, USA

Thank you for the opportunity to review this paper. I found it an insightful look at a young field of study: how journalists make use of open research. With that in mind, I have some comments, many of which are not necessarily specific suggestions for consideration as much as points the authors may want to consider in this work and efforts moving forward.

 I am comfortable with the employed search strategy for a literature review like this, but would recommend a comment that limiting searches to the literature indexed by Google Scholar will exclude a great deal of grey literature as well as writing by journalists -- who are unlikely to publish in the peer-reviewed literature.

 The manuscript does a good job of exploring just how big a barrier paywalls are, including the nuances such as articles about COVID-19 being largely available at various periods. But it is also worth noting that large publishers often make their entire libraries available to specialist reporters through organizations such as AHCJ and NASW. Eg https://m.healthjournalism.org/journal-access

 I found myself wondering whether the subjects and research approaches in preprints and OA journals markedly different from those in paywalled papers. Would that be another factor in what journalists decided to cover?

 Re: "It also does not bode well for OA journals, many of which do not (yet) have an Impact Factor because they are not indexed in Clarivate’s Web of Science database ( Bergan, 2020) or, as newer journals, may not yet be established as ‘reputable’ sources in the eyes of scientists or the journalists who report on their work." What about paywalled journals that switch (these are mentioned in passing), or the large number of OA journals that do now have IFs? Perhaps this sentence might be moved to where Schultz (2021) is discussed.

 I would like to see a bit more discussion of the watchdog role journalists can plan in holding OA work itself accountable. It's mentioned in the antidote/mitigate section but there seems to be more to say.

 Re: "For example, a small study found no significant difference in the amount of media coverage received by medRxiv preprints and peer reviewed publications about COVID-19-related therapies that were posted between February 1 and May 10, 2020 ( Jung *et al*., 2021)," it strikes me that "no significant difference" is probably still a change from before the pandemic. I might draw a more direct line between this and the fact that medRxiv did not exist until just a few months before the pandemic. Comparisons to previous pandemics and outbreaks are definitely important but may be challenging.

Is the review written in accessible language?

Yes

Are all factual statements correct and adequately supported by citations?

Yes

Are the conclusions drawn appropriate in the context of the current research literature?

Yes

Is the topic of the review discussed comprehensively in the context of the current literature?

Yes

Reviewer Expertise:

Science and medical journalism, research integrity, publishing

I confirm that I have read this submission and believe that I have an appropriate level of expertise to confirm that it is of an acceptable scientific standard.

# Response

**Published:** 2023-12-13

**Authors:** Natascha Chtena

**Affiliations:** Scholarly Communications Lab, Simon Fraser University, Vancouver, British Columbia, Canada

Thank you for taking the time to read our paper and for providing valuable comments for its improvement. 

**Comment 1: I am comfortable with the employed search strategy for a literature review like this, but would recommend a comment that limiting searches to the literature indexed by Google Scholar will exclude a great deal of grey literature as well as writing by journalists -- who are unlikely to publish in the peer-reviewed literature.**

 As suggested, we have added a sentence noting the limitations of Google Scholar in relation to grey literature: A *lthough Google Scholar indexes literature from many languages, the search algorithm is highly biased towards English-language publications ( Rovira et al., 2021); as such this language bias is a limitation of our review. **In addition, relying on Google Scholar likely excluded relevant grey literature, such as policy papers, reports, working papers, and writing by journalists.***

**Comment 2: The manuscript does a good job of exploring just how big a barrier paywalls are, including the nuances such as articles about COVID-19 being largely available at various periods. But it is also worth noting that large publishers often make their entire libraries available to specialist reporters through organizations such as AHCJ and NASW. Eg https://m.healthjournalism.org/journal-access**

 Thank you. We have added a sentence noting the access major publishers provide to specialist reporters, with the caveat that these types of partnerships are not universal: *These seemingly conflicting findings may, in part, be explained by the alternative strategies journalists have developed for accessing paywalled research articles, such as obtaining copies direct from authors ( De Dobbelaer et al., 2018; Schultz, 2023), using subscription databases to which their institutions have access ( Boss et al., 2022), and relying on free summaries or abstracts rather than complete papers ( Bray, 2019).* ***In addition, some major publishers make their libraries available to journalists who are members of specialized organizations, such as the Association of Health Care Journalists and the National Association of Science Writers (both based in the US). However, these privileges are not universal. Associations based in other countries, such as RedeComCiência (Brazilian Network of Journalists and Science Communicators), do not have the same partnerships in place, exacerbating asymmetries between the Global North and South.***

**Comment 3: I found myself wondering whether the subjects and research approaches in preprints and OA journals markedly different from those in paywalled papers. Would that be another factor in what journalists decided to cover?**

 It could be that research available as a preprint or an OA journal article differs from research published in a closed access journal, but we don’t feel there is a strong enough body of evidence to substantiate this claim. In fact, there is evidence that most preprints get published (Abdil & Blekhman, 2019; Fraser et al., 2020) and that those that do are relatively similar to the final journal article (e.g., Brierley et al., 2022). In addition, many preprint servers do not accept non-traditional outputs, and they also screen for format/layout, meaning that they won't accept submissions that don't follow the traditional scholarly article structure (Malički et al., 2020). That is, they won't accept things that are "markedly different" from journal articles. We also are not aware of any research suggesting any established differences between OA journals and paywalled ones, as the boundaries have become so blurry in recent years. 

 Abdill, R. J., & Blekhman, R. (2019). Tracking the popularity and outcomes of all bioRxiv preprints. eLife, 8, e45133. https://doi.org/10.7554/eLife.45133

 Brierley, L., Nanni, F., Polka, J. K., Dey, G., Pálfy, M., Fraser, N., & Coates, J. A. (2022). Tracking changes between preprint posting and journal publication during a pandemic. PLOS BIOLOGY, 20(2), e3001285. https://doi.org/10.1371/journal.pbio.3001285

 Fraser, N., Momeni, F., Mayr, P., & Peters, I. (2020). The relationship between bioRxiv preprints, citations and altmetrics. Quantitative Science Studies, 1(2), 618–638. https://doi.org/10.1162/qss_a_00043

 Malički, M., Jerončić, A., ter Riet, G., Bouter, L. M., Ioannidis, J. P. A., Goodman, S. N., & Aalbersberg, Ij. J. (2020). Preprint servers’ policies, submission requirements, and transparency in reporting and research integrity recommendations. JAMA, 324(18), 1901–1903. https://doi.org/10.1001/jama.2020.17195

**Comment 4:** **"It also does not bode well for OA journals, many of which do not (yet) have an Impact Factor because they are not indexed in Clarivate’s Web of Science database ( Bergan, 2020) or, as newer journals, may not yet be established as ‘reputable’ sources in the eyes of scientists or the journalists who report on their work." What about paywalled journals that switch (these are mentioned in passing), or the large number of OA journals that do now have IFs? Perhaps this sentence might be moved to where Schultz (2021) is discussed.**

 Per your suggestion, we have updated this section to provide a more nuanced discussion of OA journals and IFs: *It also does not bode well for **some** OA journals, which do not (yet) have an Impact Factor because they are not indexed in Clarivate’s Web of Science database ( Bergan, 2020) or, as newer journals, may not yet be established as ‘reputable’ sources in the eyes of scientists or the journalists who report on their work. **Of course, these same reservations may apply to some closed access journals as well, and may not be relevant to major OA journals with high Impact Factors and recognized brands, such as PLOS Medicine or Nature Communications. Still, **exploratory research suggests that some journalists are “more suspicious of open access journals, believing they lacked a credible review process” ( Van Witsen & Takahashi, 2021, p. 10).*

**Comment 5:**** **I would like to see a bit more discussion of the watchdog role journalists can plan in holding OA work itself accountable. It's mentioned in the antidote/mitigate section but there seems to be more to say.**

 We have expanded on this in the section “The argument for OS-based journalism”:

*Similarly, although health and science journalists fulfill some traditional journalistic roles—such as watchdog (holding powerful scientific or pharmaceutical institutions to account) and agenda setter (driving attention to new trends, issues, and findings in research)—they also play additional roles such as the civic educator, using their skills to teach audiences about the nature of scientific research and its limits and risks ( Fahy & Nisbet, 2011). These roles and functions, while not always consistently performed in practice, **are important for ensuring that the growing trend towards openness in science supports the interests of society and the integrity of the scholarly record. For example, science journalists have published nuanced, critical coverage of recent OS-related controversies, such as the use of predatory practices among major OA publishers (Brainard, 2023; Kolata, 2017), flawed preprint studies (Miller, 2022; Bartlett, 2023), and the high cost of article processing charges associated with OA publishing (Ansede, 2023). Similarly, Retraction Watch—a blog and database founded and managed by science and health journalists—maintains a running list of retracted COVID-19 articles, including OA articles and preprints, and regularly features news about problematic research practices, including fraud, plagiarism, and predatory publishing in both closed and open science.***

***Science journalists’ ability to call attention to pernicious aspects of OS, while simultaneously helping publics take advantage of its benefits, makes them ideally positioned to help make** “scientific knowledge openly available, accessible and reusable for everyone, to increase scientific collaborations and sharing of information for the benefits of science and society, and to open the processes of scientific knowledge creation, evaluation, and communication to societal actors beyond the traditional scientific community” ( UNESCO, 2021, p. 6).*

**Comment 6: Re: "It also does not bode well for OA journals, many of which do not (yet) have an Impact Factor because they are not indexed in Clarivate’s Web of Science database ( Bergan, 2020) or, as newer journals, may not yet be established as ‘reputable’ sources in the eyes of scientists or the journalists who report on their work." What about paywalled journals that switch (these are mentioned in passing), or the large number of OA journals that do now have IFs? Perhaps this sentence might be moved to where Schultz (2021) is discussed.**

 Jung et al. (2021) found that preprints were covered in a median of 1.5 news stories while peer reviewed publications were covered in a median of 1 news story. However, we are hesitant to draw attention to this difference given it was non significant (p value of 0.70) and the sample was very small (n=106), focused on a narrow topic, and likely not representative of coverage of other COVID-19 or medRxiv preprints. 

 However, we have addressed your concern about distinguishing the effects of the launch of medRxiv on preprint coverage from the effects of the onset of the pandemic by adapting the following sections. We cite research that was not yet published when we conducted our initial review: 

*Similarly, coverage of preprints in German news outlets was relatively low before the pandemic, but surged in 2020 and 2021 ( Simons & Schniedermann, **2023). Finally, a study found that preprints were featured in less than 2% of media coverage of research before the pandemic, but that this proportion surged to almost 4% after the onset of COVID-19 (Fleerackers et al., 2023). Moreover, this surge appeared to be driven entirely by COVID-19 preprints, as the launch of the medical preprint server medRxiv in 2019 had little or no effect on rates of preprint coverage. **Some journalists describe this widespread adoption of COVID-19 preprints as a “paradigm shift” that is likely to persist post-pandemic ( Fleerackers et al., 2022). Scholars have made similar claims that the recent coverage of preprints represents a long-term “cultural shift” in journalism ( Fraser et al., 2021, p. 18; Stollorz, 2021; van Schalkwyk & Dudek, 2022).*

*In addition, although journalists feel positive about open research in general—even more now than before the pandemic—they remain more skeptical of preprints than OA journal publications ( Schultz, 2023).  Moreover, it is possible that the volume of preprint-coverage varies across geographies, media outlets, and individual journalists. For example, Massarani et al. (2021a) found that journalists in the Asia/Pacific region were among the most likely to use preprints, whereas those in African and Middle Eastern countries were among the least likely. **In addition, Fleerackers et al. (2023) found little or no change in the coverage of non-COVID-19 preprints during the pandemic period, suggesting that journalists’ embrace of COVID-19 preprints may not extend to preprints on other topics, nor those posted during less urgent crisis contexts.***

# Reviewer report: Reviewer response for version 1

**DOI:** 10.5256/f1000research.146721.r174170

**Published:** 2023-06-06

**Authors:** Cameron Neylon

**Affiliations:** 1 Curtin University, Perth, Western Australia, Australia

This is a timely and important review of the relationship between open access and dissemination practices and science journalism during the COVID-19 pandemic. It is particularly valuable in identifying gaps in the literature with respect to preprints, journalism and their risks and benefits.

 My expertise is not in studies of science journalism so additional expertise may be required to ensure the comprehensive coverage of the review. From the perspective of open science practices the review covers the core aspects of importance. I would find it helpful to have a table or dataset that categorizes or lists the identified outputs as well as the relevant search terms but this is a minor issue.

 Minor point: Introduction paragraph 4 "...a review of a review of..." is presumably a duplication? If not then maybe rephrasing will help.

Is the review written in accessible language?

Yes

Are all factual statements correct and adequately supported by citations?

Yes

Are the conclusions drawn appropriate in the context of the current research literature?

Yes

Is the topic of the review discussed comprehensively in the context of the current literature?

Yes

Reviewer Expertise:

Open science, research evaluation, scholarly publishing

I confirm that I have read this submission and believe that I have an appropriate level of expertise to confirm that it is of an acceptable scientific standard.

# Response

**Published:** 2023-12-13

**Authors:** Natascha Chtena

**Affiliations:** Scholarly Communications Lab, Simon Fraser University, Vancouver, British Columbia, Canada

Thank you for taking the time to read our paper and for your valuable feedback. 

**Comment 1: I would find it helpful to have a table or dataset that categorizes or lists the identified outputs as well as the relevant search terms but this is a minor issue.**

 Thank you for this suggestion. All of the reviewed outputs are already included in the bibliography and we have not included specific search terms because we do not wish to imply that the search was systematic. We also employed a snowball search approach to identify relevant works by using the bibliography or reference list of already identified papers. As such, while we see the value of this suggestion for a systematic review or meta-analysis, we do not feel it is appropriate or necessary here. 

**Comment 2:** **Introduction paragraph 4 "...a review of a review of..." is presumably a duplication? If not then maybe rephrasing will help.**

 It was indeed a duplication and has now been corrected. Thank you for catching that.
