# Supplementary material for: Making science public: a review of journalists’ use of Open Science research
Source: F1000Res. 2023 May 18;12:512. [Version 1] doi: 10.12688/f1000research.133710.1 (PMC10618641; doi:10.12688/f1000research.133710.1)
Supplement: Peer review history

# Reviewer report: Reviewer response for version 1

**DOI:** 10.5256/f1000research.146721.r214223

**Published:** 2023-11-08

**Authors:** Henrik Karlstrøm

**Affiliations:** 1 Nordic Institute for Studies of Innovation, Research and Education, Oslo, Norway

Given that increased public engagement with scientific research is an impetus of the Open Science movement itself, the research question of this paper is highly relevant. The authors do a good job of establishing the ways in which brokerage between science and journalism can improve public understanding of the scientific process. I find the paper well-structured, well-argued and discussing a topic of potentially high import.

 The paper is quite limited in scope. The authors note that there are few empirical investigations of science journalists' engagement with OS literature, and state that "this review mainly covers research and theoretical contributions that discuss the intersections of OS and SJ tangentially or as a secondary concern, rather than a primary focus". It is good that the authors acknowledge the limitations of reviewing a literature that is only tangentially concerned with the question at hand. You can only work with what is available, of course, and the authors do note that an empirical investigation of journalists' use of code and data is planned in the future. However, this limitation to conceptual discussions obviously affects the scope of the findings claimed by the paper.

 Given the lack of empirical data, the authors are correct to call this a review of existing literature. There is a discrepancy between how this is framed in the abstract and in the key recommendations. The abstract states: "We find that, despite journalists’ potential to act as critical brokers of open access knowledge, their use of open research outputs is hampered by an overreliance on traditional criteria for evaluatingscientific quality; concerns about the trustworthiness of open research outputs; and challenges using and verifying the findings." However, these are not really findings, given that the literature reviewed does not directly address these questions. A better framing would have been to focus on the gaps identified in the literature, as these are amply demonstrated in the paper.

 Overall, this is a useful overview of the literature that exists at the crossroads of science journalism, open science and COVID research. It constitutes a taking stock of the current situation and a roadmap for further investigation into the factors that shape decisions by non-scientists who are trying to navigate the somewhat insular world of academic publishing.

Is the review written in accessible language?

Yes

Are all factual statements correct and adequately supported by citations?

Yes

Are the conclusions drawn appropriate in the context of the current research literature?

Yes

Is the topic of the review discussed comprehensively in the context of the current literature?

Yes

Reviewer Expertise:

Scientific publishing, metascience, bibliometrics

I confirm that I have read this submission and believe that I have an appropriate level of expertise to confirm that it is of an acceptable scientific standard.
